# Investigation of the Impact of Clinker Grinding Conditions on Energy Consumption and Ball Fineness Parameters Using Statistical and Machine Learning Approaches in a Bond Ball Mill

**DOI:** 10.3390/ma18133110

**Published:** 2025-07-01

**Authors:** Yahya Kaya, Veysel Kobya, Gulveren Tabansiz-Goc, Naz Mardani, Fatih Cavdur, Ali Mardani

**Affiliations:** 1 Department of Civil Engineering, Bursa Uludag University, Nilüfer 16059, Bursa, Turkey; yahyakaya00@gmail.com (Y.K.); v.kobya@gmail.com (V.K.); 2 Faculty of Engineering, Architecture and Design, Department of Industrial Engineering, Mudanya University, Mudanya 16940, Bursa, Turkey; gulverentabansiz@gmail.com; 3 Department of Mathematics Education, Bursa Uludag University, Nilüfer 16059, Bursa, Turkey; nazmardani@uludag.edu.tr; 4 Department of Industrial Engineering, Bursa Uludag University, Nilüfer 16059, Bursa, Turkey; fatihcavdur@uludag.edu.tr

**Keywords:** machine learning, cement grinding optimization, gradient boosting, ridge regression, support vector regression

## Abstract

This study explores the application of machine learning (ML) techniques—gradient boosting (GB), ridge regression (RR), and support vector regression (SVR)—for estimating the consumption of energy (CE) and Blaine fineness (BF) in cement clinker grinding. This study utilizes key clinker grinding parameters, such as maximum ball size, ball filling ratio, clinker mass, rotation speed, and number of revolutions, as input features. Through comprehensive preprocessing, feature selection methods (mutual info regression (MIR), lasso regression (LR), and sequential backward selection (SBS)) were employed to identify the most significant variables for predicting CE and BF. The performance of the models was optimized using a grid search for hyperparameter tuning and validated using k-fold cross-validation (k = 10). The results show that all ML methods effectively estimated the target parameters, with SVR demonstrating superior accuracy in both CE and BF predictions, as evidenced by its higher R^2^ and lower error metrics (MAE, MAPE, and RMSE). This research highlights the potential of ML models in optimizing cement grinding processes, offering a novel approach to parameter estimation that can reduce experimental effort and enhance production efficiency. The findings underscore the advantages of SVR, making it the most reliable method for predicting energy consumption and Blaine fineness in clinker grinding.

## 1. Introduction

The global climate crisis and its irreversible impacts have highlighted the role of cement production, which significantly contributes to greenhouse gas emissions and energy consumption [1,2,3,4,5]. Two main strategies have emerged to mitigate these effects. The first involves developing alternative binders and supplementary cements [6,7,8,9,10,11,12,13] and exploring their usability [14,15,16,17]. The second focuses on optimizing the clinker grinding process, a major energy consumer in cement production [18,19,20,21,22,23].

Grinding efficiency is influenced by parameters such as mill type and size, grinding medium properties (e.g., size, shape, and material), rotational speed, and the type, quantity, and fineness of the material being ground [20,22,24]. Ball mills, commonly used in the final grinding stage, accommodate both wet and dry materials. The grinding medium, typically cast steel, cast iron, or forged steel, plays a critical role in this process [25].

Grinding operates through three mechanisms: (i) impact or compression from forces perpendicular to the particle surface, (ii) fragmentation from oblique forces, and (iii) abrasion from parallel forces [20,21,24]. These forces exceed the particles’ modulus of elasticity, causing deformation and fracture [20]. While the motion of the grinding medium within the mill is complex, simulation methods provide insights into their general behavior [26,27].

The mill’s critical speed (ωc) defines the point at which balls are suspended, with mills typically operating at 65–82% of this speed, occasionally reaching up to 90% [20,25]. Ball size is another key factor; larger balls reduce the feed size through impact and compression [18,20], while smaller balls enhance fineness via abrasion [20,25]. For optimal grinding efficiency, a mix of ball sizes is recommended [20,21,22]. Since the grinding medium and load behavior directly affect product size, energy consumption, and costs, detailed analysis and optimization are essential for process efficiency.

Given the numerous factors affecting grinding conditions, experimental investigations are often costly and time-consuming [19,20,21,24,28,29]. Consequently, modeling and regression techniques are widely applied to streamline optimization [22,30,31,32].

In recent years, machine learning (ML) approaches have increasingly been used to predict outputs in material processes, offering significant cost and time savings by replacing repetitive physical experiments with computational models trained on historical data. Studies have demonstrated the effectiveness of various ML methods, including gradient boosting (GB), ridge regression (RR), support vector regression (SVR), artificial neural networks (ANN), decision trees (DT), extra trees (ET), gene expression programming (GEP), and random forests (RF). Boosting algorithms like light gradient boosting (LGB) and extreme gradient boosting (XGB) have also gained prominence. For instance, GB and its derivatives have been applied to predict concrete compressive strength [33,34], while models like RF, ANN, and SVM have been used for sustainable high-performance concrete predictions [35] and phase-change material composites [36]. These advancements highlight ML’s potential to enhance the efficiency and accuracy of material property predictions.

Various machine learning techniques have been employed in prior studies for prediction tasks. For instance, Belalia et al. [37] and Yaman [38] utilized ANN to forecast self-compacting concrete’s properties and mix content, respectively. Han et al. [39], Zhang et al. [40], and Mai et al. [41] applied RF to predict compressive strength for high-performance concrete, lightweight self-compacting concrete, and ground-granulated blast-furnace slag concrete. Farooq et al. [42] and Iftikhar et al. [43] used GEP and RF for similar predictions, while Sarir et al. [44] analyzed the bearing capacity of concrete-filled steel tube columns using ANN and GEP. Shahmansouri et al. [45] employed GEP to estimate the compressive strength and electrical resistivity of eco-friendly concrete with natural zeolite, Aslam et al. [46] did the same for high-strength concrete, and Shah et al. [47] analyzed the compressive and tensile strength of fly ash concrete. Zeini et al. [48] and Zhou et al. [49] predicted the strengths of geopolymer-stabilized clayey soils and geopolymer concrete using RF and DT. Chou et al. [50] modeled concrete compressive strength with MART and ANN, while Cheng and Cao [51] combined MARS and ANN for similar predictions. Kaveh et al. [52] employed M5 Tree and MARS to predict compressive strength and fresh-state properties, and Nasr et al. [53] integrated PSO-LightGBM to evaluate mechanical and electrical properties of roller-compacted concrete with ceramic waste under freeze conditions.

Despite extensive machine learning applications in predicting concrete properties, energy consumption and Blaine fineness remain unexplored, as observed in the literature. This study significantly contributes by successfully employing GB, RR, and SVR models to estimate these parameters.

## 2. Methods

### 2.1. Database Description and Preprocessing

**Figure 1:**
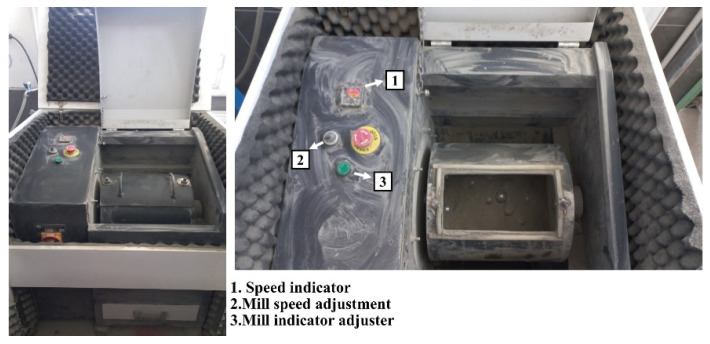
Ball mill used in the study.

The data in this study were derived from the experimental results of Kaya et al. [22]. A laboratory mill (MicroAnalysis Inc., 2023; Ankara, Türkiye) with a 5 kg capacity and 1.5 kW motor power, depicted in Figure 1, was used for clinker grinding.

The mill feed, comprising 96% clinker and 4% gypsum, was set at 2, 3, and 4 kg. Nine ball diameters were employed in six different distributions, with Distribution 6 chosen per the Bond Standard, while the other five were selected based on literature guidelines. The mill’s rotation speed was set at 40, 55, and 70 rpm, corresponding to 50%, 70%, and 90% of its critical speed. The influence of these parameters on grinding efficiency was analyzed using two approaches. In the first approach, the Blaine fineness of cement samples was measured after 4000, 5000, and 6000 cycles. In the second approach, the number of grinding cycles required to achieve a target Blaine fineness of 3700 ± 100 g/cm^2^ was recorded. Thus, a total of 216 results were obtained as a result of the experimental study, which was conducted in two stages.

The energy consumed by the mill under each grinding condition (ball distribution, feed mass, and speed) was calculated according to Equation (1).

Eg = (220 × To × A × 1000)/(m × Tg) (1)

In this context, Eg denotes the grinding energy (kWh/ton), To represents the grinding time (hours), A is the amperage, m is the feed amount (kg), and Tg is the mill factor, a constant provided by the manufacturer with a value of 4. Each grinding process was conducted in triplicate to ensure consistency, and the results were averaged to obtain representative values. This approach allowed for the assessment of experimental repeatability and enhanced the reliability of the data presented. The limitations of this study arose from the discrepancies between laboratory ball mill conditions (e.g., ball distribution, feed amount, rotation speed) and the operational conditions of industrial mills.

In this study, univariate outlier analysis was performed to identify outliers. Each variable was examined using boxplot and pair plot methods as part of the univariate outlier analysis. The dataset was thoroughly evaluated both statistically and visually, with no outliers detected. Additionally, the dataset was checked for missing values prior to modeling, and no missing data were found in any of the input or output variables. Finally, in order to ensure comparability among the variables and to improve model performance, all input features were standardized using Z-score normalization. Table 1 presents the summary statistics of input and outcome variables for CE, while Table 2 provides the same for BF.

**Figure 2:**
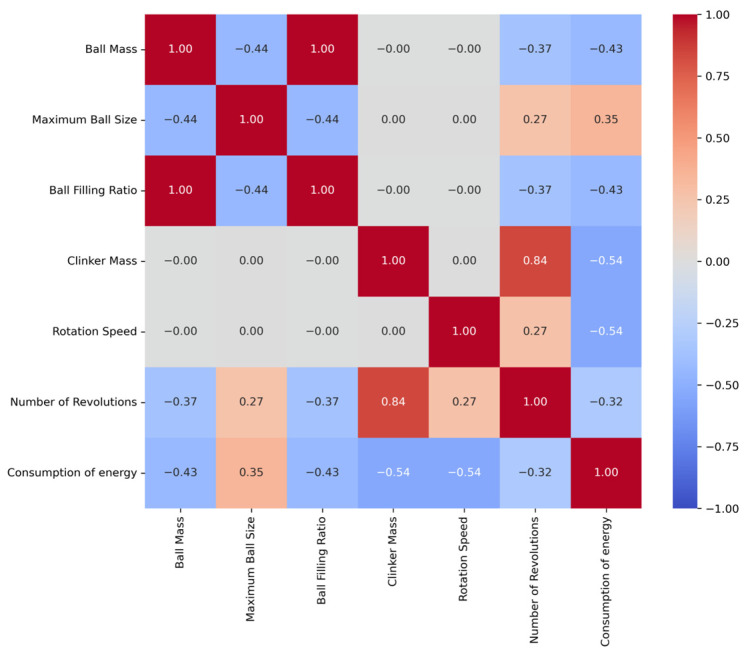
Pearson’s correlation coefficient between any two variables (CE).

**Figure 3:**
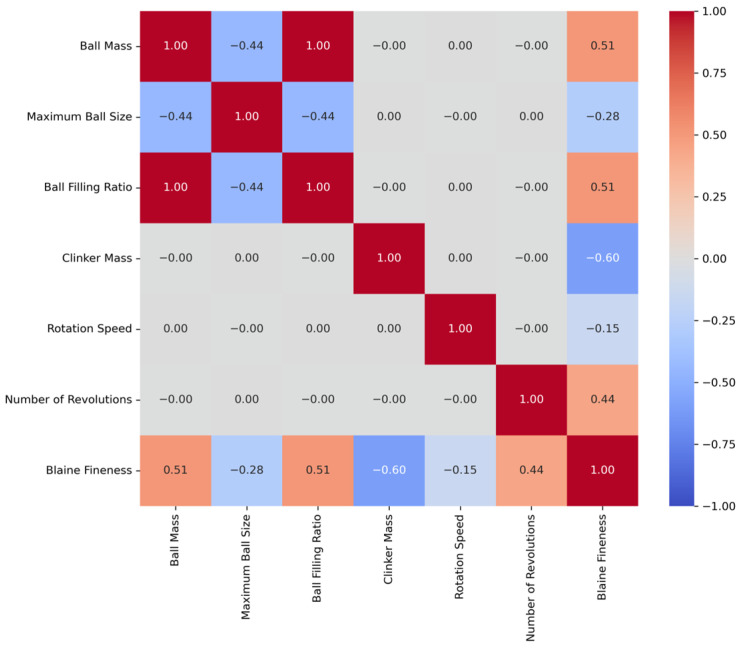
Pearson’s correlation coefficient between any two variables (BF).

These tables include basic statistical measures such as the mean, standard error, median, mode, standard deviation, range, minimum, and maximum for each variable. Furthermore, Figure 2 and Figure 3 illustrate the pairwise Pearson correlation coefficients for the CE and BF variables, respectively.

**Figure 4:**
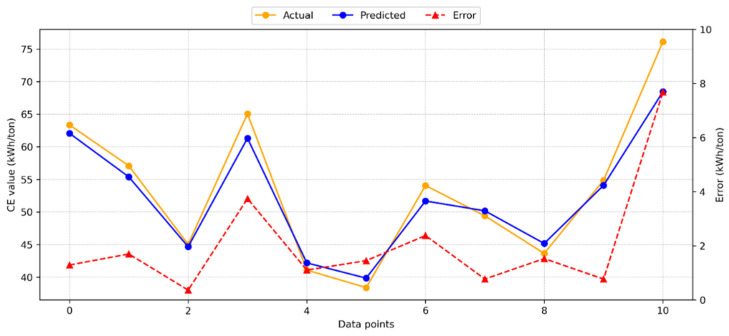
Dispersal of the GB model’s estimated, actual, and absolute error values for CE.

Analysis of the correlation matrices reveals significant relationships among various variables for both CE and BF. A notable finding for CE is the strong positive correlation between clinker mass, number of revolutions, and CE value (0.54). Similarly, Figure 4 highlights a positive correlation with ball mass (0.51) and a negative correlation with clinker mass (−0.60). These results indicate that ball mass and clinker mass are directly related to BF, with the BF value decreasing as clinker mass increases. In this study, GB, RR, and SVR were employed to model the effects of grinding process parameters on CE and BF. RR was applied to identify linear relationships among the variables and to address multicollinearity issues. In contrast, SVR and GB, being more flexible and robust, were used to capture potential nonlinear interactions and threshold behaviors that might not be revealed through conventional correlation analysis. These models are capable of learning complex patterns that linear methods may overlook, thus improving both predictive accuracy and generalizability. Therefore, the combined use of linear and nonlinear modeling approaches provides a more comprehensive and reliable understanding of how grinding parameters influence CE and BF. The dataset was split into a training set and a test set at a ratio of 80% to 20%, respectively, and model training was conducted. The scikit-learn library was extensively used in this study for implementing all machine learning models, performing feature selection, optimizing hyperparameters, and conducting model validation through k-fold validation.

### 2.2. Feature Selection

Feature selection methods offer benefits such as reduced data collection costs and improved interpretability of classification models [54]. These methods are generally classified into three categories: filter, wrapper, and embedded methods [55,56]. The advantages and limitations of these approaches are extensively reviewed by Ladha and Deepa [55], Saeys et al. [57] and Bolon-Canedo et al. [58]. This study employs three feature selection methods: mutual info regression (MIR), lasso regression (LR), and sequential backward selection (SBS).

MIR was first applied to evaluate feature reliability by quantifying mutual information between two variables using probability density functions p(x), p(y), and p(x, y). This ensures that the selected features exhibit high levels of mutual information. Subsequently, the top (m) features are sequentially chosen. LR, another method, effectively reduces dimensionality and complexity by identifying significant correlation coefficients while eliminating irrelevant features. Lastly, SBS, which operates via backward elimination, begins with the full set of attributes and iteratively removes redundant ones. This method is particularly effective for datasets with a large number of attributes [55]. The selected and excluded features for CE and BF prediction, based on different selection methods, are detailed in Table 3.

For the prediction of CE, the MIR method excluded ball mass, maximum ball size, and ball filling ratio, while the LR model omitted the same variables. The SBS approach excluded maximum ball size, ball filling ratio, and the number of revolutions. Among the models, MIR and LR demonstrated superior predictive performance when using clinker mass, rotation speed, and the number of revolutions as input variables.

In the prediction of BF, the MIR method excluded maximum ball size, whereas LR eliminated ball mass and rotation speed. Similarly, SBS removed maximum ball size, aligning with the MIR selection. The most accurate predictions were obtained using SBS and MIR, incorporating ball mass, ball filling ratio, clinker mass, rotation speed, and number of revolutions as input features.

As noted from the aforementioned definitions of the feature selection methods used in this study, the exclusion of some of the attributes does not necessarily imply the irrelevance of the corresponding features (as might also be the case for some of the attributes in this study), but rather their redundancy due to various issues, such as multicollinearity or low variance caused by including the attributes which are highly correlated with the others and the attributes with low variances, respectively. Such attributes are usually excluded during the feature selection process as they might not provide added value for prediction. In addition, since the feature selection methods employed in the study (MIR, LR and SBS) rely on different criteria, some features may have been significant only for one variable. This variation reflects the diverse information requirements and influences mechanisms of each variable in the models.

### 2.3. Hyperparameter Tuning and Optimization

Hyperparameters are a set of parameters used to optimize the learning process in machine learning models. In supervised machine learning (ML) models, including regression and classification, selecting appropriate hyperparameters is a critical step in model training [59]. Hyperparameter values can be determined using default settings from ML packages or through a trial-and-error process. However, the trial-and-error approach can be time-consuming and labor-intensive [60]. To save time and resources, hyperparameter optimization and tuning techniques are often employed.

Choosing the optimal hyperparameters is essential for minimizing model error and achieving the highest accuracy [61]. In this study, the Grid Search method was used to identify the ideal hyperparameters. Cross-validation was applied to assess the model’s effectiveness by ensuring that it provided a reliable and accurate representation of its ability to generalize to new data [62]. For each ML technique, a set of adjustable hyperparameters and their respective ranges were defined. The optimal hyperparameters, identified through Grid Search, were then evaluated using k-fold validation (k = 10). In k-fold validation, the training dataset is divided into (k) parts, with (k − 1) parts used for training and the remainder used for validation [63]. During this process, the mean absolute error (MAE), root mean squared error (RMSE), mean absolute percentage error (MAPE), and the coefficient of determination (R^2^) values were computed for each trial, with results averaged over ten iterations. To estimate overall model performance, all possible combinations of hyperparameters were tested, and the results for each level were evaluated. Table 4 and Table 5 display the ranges and optimal values of the hyperparameters for energy consumption and Blaine fineness predictions, respectively, while Table 6 and Table 7 show the optimal hyperparameters and k-fold validation results for these predictions.

In the model selection process, emphasis was placed not only on average performance metrics but also on the overall generalizability and robustness of the models. As shown in Table 6 and Table 7, the 10-fold cross-validation results—particularly in terms of R^2^ scores—demonstrate that both the gradient boosting (GB) and support vector regression (SVR) models consistently achieved strong predictive performance across different data partitions.

In the energy consumption estimation task, SVR attained the highest average R^2^ value of 0.92, while GB followed closely with a competitive average of 0.86. For Blaine fineness prediction, all three models produced similarly high average R^2^ values, with both GB and ridge regression (RR) yielding 0.88 and SVR slightly outperforming them with a score of 0.89. Notably, SVR exhibited the lowest variance across folds in both tasks, underscoring its robustness and consistent behavior across varying subsets of the data. GB, on the other hand, achieved exceptionally high R^2^ scores in several folds, reaching values as high as 0.99, which illustrates its strong ability to model complex, nonlinear relationships and its high generalization capacity. Although RR demonstrated comparatively lower predictive power, it remains valuable due to its interpretability and stable performance, particularly in settings where linear assumptions are reasonable. Consequently, the final model selection was guided not only by mean accuracy, but also by the models’ consistency across folds and their potential for generalization—ensuring the methodological soundness and practical relevance of the proposed approach. Additionally, an ensemble modeling approach using simple averaging and weighted averaging was implemented to enhance prediction performance. Detailed results for CE and BF are provided in Appendix A Table A1 and Table A2, respectively, for interested readers.

### 2.4. Description of Employed Techniques

In this study, ridge regression (RR), support vector regression (SVR), and gradient boosting (GB) were selected due to the limited size of the dataset and the need to effectively model both linear and nonlinear relationships between input features and the target variable. RR, as a linear model, was included as a baseline, particularly useful in cases of multicollinearity. SVR was preferred for its strong capability in capturing nonlinear patterns, while GB was chosen for its high accuracy through sequential learning, even with small datasets.

At the initial stage of the study, other algorithms such as random forest, XGBoost, and neural networks were also tested. However, these models did not yield significant performance improvements over the selected ones. Moreover, they increased model complexity, reduced interpretability, and posed a higher risk of overfitting given the dataset size. Therefore, based on the scope and to maintain clarity and relevance of the results, the focus was placed on the three models that performed best overall.

#### 2.4.1. Gradient Boosting Regressor

Gradient boosting (GB) is an ensemble learning technique commonly used to address regression problems [63]. It works by iteratively refining predictions through the combination of multiple “weak” learners. In each iteration, the algorithm aims to minimize the discrepancy between the actual target values and the ensemble predictions by training each subsequent learner to estimate the negative gradient of a loss function relative to the current ensemble estimates. The outputs of all learners are then aggregated to form the final prediction. A key hyperparameter in GB is the learning rate, which controls the step size for adjusting each weak learner. A smaller learning rate typically ensures more stable convergence and enables the model to better capture complex relationships within the regression data [64]. GB is a powerful machine learning technique widely used across various applications due to its effectiveness in managing complex input–output relationships and minimizing residual errors.

#### 2.4.2. Ridge Regressor

For a multiple linear regression model to make a successful prediction, certain assumptions must be fulfilled. One of these assumptions is the absence of multicollinearity among the explanatory variables in the model. However, alternative methods have been developed to solve this problem in the presence of multicollinearity. The most important of these methods is the ridge regression (RR) method, which estimates the parameter coefficients in a biased manner without excluding the variables in the model. By taking into account all variables that should be included in the model, RR provides parameter estimates with lower variance than the estimates obtained by the least squares method and aims to reduce the effect of variables that should not be included in the model [65]. This approach was developed specifically to mitigate the effects of the multicollinearity problem.

#### 2.4.3. Support Vector Regressor

The support vector machine (SVM) is a supervised machine learning technique introduced by Vapnik [66], grounded in the Vapnik–Chervonenkis theory. Initially designed to address classification and regression problems, SVM was later adapted as support vector regression (SVR) to enhance model prediction accuracy [67]. SVR works by minimizing prediction errors while determining the optimal fitting function for the training data. Additionally, it optimizes the smoothness of the function, which helps to reduce the likelihood of the model becoming trapped in local minima during the training process [68].

#### 2.4.4. Performance Evaluation of Models

The performance of the developed machine learning models was assessed using statistical parameters such as R^2^, MAE, RMSE, and MAPE. The R^2^ score reflects the accuracy of the models, quantifying the discrepancy between predicted values and actual targets [69]. A lower R^2^ value, closer to zero, suggests a higher level of bias, while a value closer to one indicates a lower degree of bias. Smaller errors derived from these statistical tests signify greater model accuracy. The statistical evaluation of model accuracy was conducted using Equations (1)–(3), where *n* represents the number of data points, $P_{i}$ is the predicted model result, and $E_{i}$ is the actual test result.

(2) $$MAE=\frac{1}{n}\sum_{i=1}^{n}\left|P_{i}-E_{i}\right|$$

(3) $$RMSE=\sqrt{\sum_{i=1}^{n}\frac{{\left(P_{i}-E_{i}\right)}^{2}}{n}}$$

(4) $$MAPE=\frac{100\%}{n}\sum_{i=1}^{n}\frac{\left|P_{i}-E_{i}\right|}{E_{i}}$$

## 3. Results and Analysis

### 3.1. Consumption of Energy Estimation

#### 3.1.1. Gradient Boosting Model for CE

Figure 4 presents the results from applying the gradient boosting (GB) model to calculate CE.

The GB model demonstrated high accuracy, with a minimal deviation between the predicted and actual test results. The R^2^ value of 0.9319 indicates a strong agreement between the test and model predictions. Additionally, Figure 4 illustrates the distribution of predicted values, actual values, and errors for the GB model. The maximum observed error was 7.67 kWh/ton, while the average error was 2.07 kWh/ton. These error results suggest that the GB model provides a reliable estimate for CE.

#### 3.1.2. Ridge Regression Model for CE

**Figure 5:**
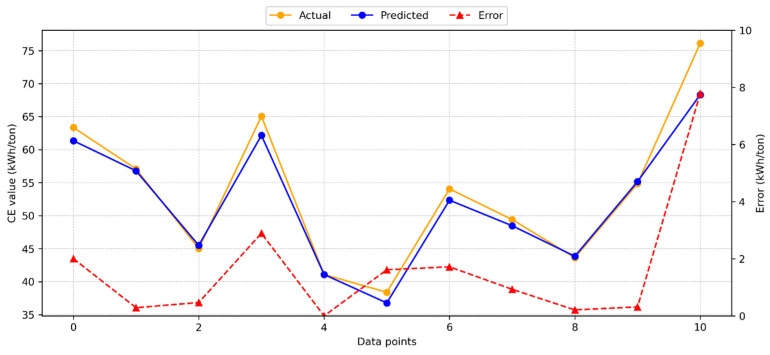
Dispersal of the RR model estimated, actual, and absolute error values for CE.

Figure 5 displays the results from the RR model in predicting CE.

Compared to the GB model, the RR model produced nearly identical results, with minimal variance between the test and model predictions. The RR model achieved an R^2^ score of 0.939, indicating a high level of accuracy. Figure 5 also illustrates the distribution of predicted values, actual values, and errors generated by the RR method. The maximum observed error was 7.78 kWh/ton, while the average error was 1.65 kWh/ton. These results suggest that the RR model provides a reliable and accurate estimate for CE.

#### 3.1.3. Support Vector Regression Model for CE

**Figure 6:**
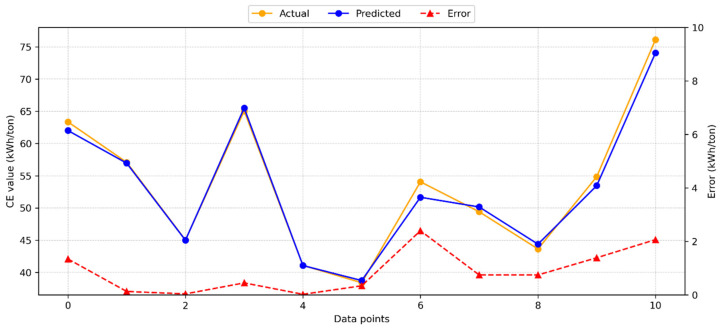
Dispersal of the SVR model estimated, actual, and absolute error values for CE.

Figure 6 presents the results of using the SVR method to estimate CE, showing the model’s predictions, actual values, and the distribution of error values.

The SVR model outperformed the GB and RR models, exhibiting the highest accuracy with an R^2^ score of 0.988. This score indicates a strong correlation between the predicted and actual values. The distribution of error values reveals that the maximum error was 2.39 kWh/ton, while the average error was 0.87 kWh/ton. These results demonstrate that the SVR method provides superior accuracy and minimal variability in predictions compared to the GB and RR models.

### 3.2. Blaine Fineness Prediction

#### 3.2.1. Gradient Boosting Model for BF

**Figure 7:**
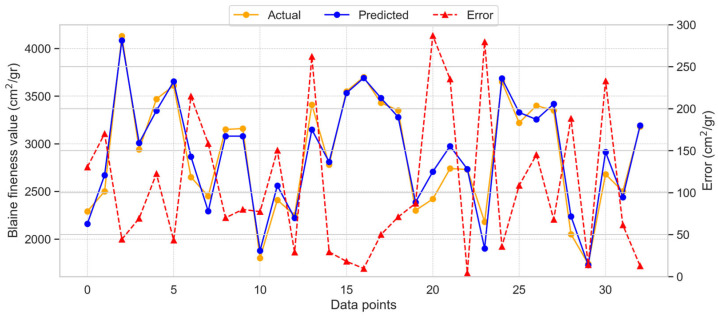
Dispersal of the GB model estimated, actual, and absolute error values for BF.

Figure 7 displays the results obtained by applying the GB model to predict BF.

The model provided accurate predictions with minimal deviation between the test and predicted results, yielding an R^2^ score of 0.946. This indicates a satisfactory level of agreement between the predicted and actual values. The distribution of model predictions, actual values, and error values is shown in Figure 7. The maximum observed error was 287.0 cm^2^/gr, while the average error was 107.85 cm^2^/gr. These error results suggest that the GB model delivers a reasonable estimate for BF.

#### 3.2.2. Ridge Regression Model for BF

**Figure 8:**
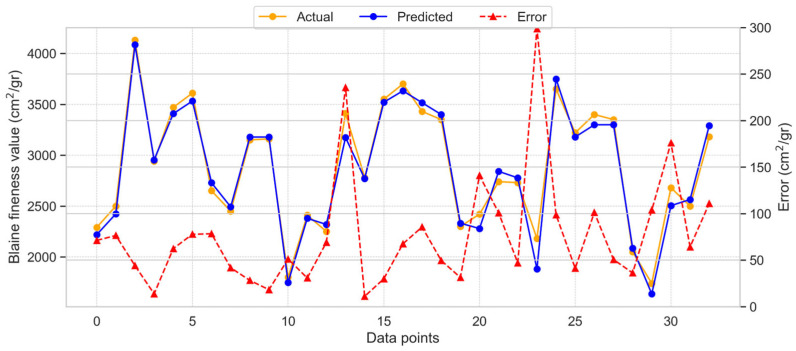
Dispersal of the RR model estimated, actual, and absolute error values for BF.

Figure 8 presents the results of the RR model applied to predict BF.

Compared to the GB model, the RR model provided more accurate results with minimal variance between the test and predicted values. The RR model achieved an R^2^ score of 0.972, indicating a high level of agreement between the predicted and actual values. Figure 8 shows the distribution of model predictions, actual values, and error values. The maximum observed error was 298.53 cm^2^/gr, while the average error was 77.06 cm^2^/gr. These results highlight the improved accuracy of the RR model in estimating BF.

#### 3.2.3. Support Vector Regression Model for BF

**Figure 9:**
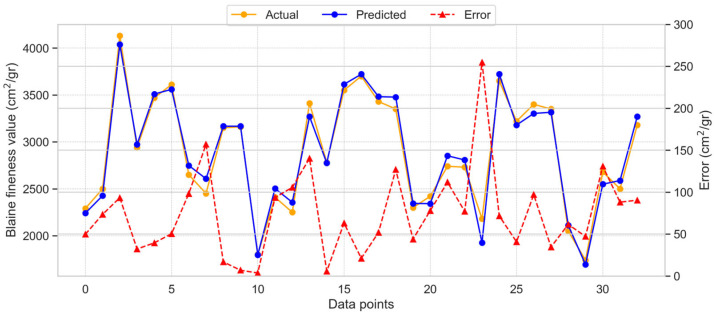
Dispersal of the SVR model estimated, actual, and absolute error values for BF.

Figure 9 presents the results of using the SVR method to estimate BF.

The SVR provided more accurate results compared to both the GB and RR models, exhibiting the lowest degree of variability between the actual and model-estimated results. The R^2^ score of 0.977 for the SVR model indicates its high accuracy. Figure 9 illustrates the distribution of model estimates, actual values, and error values for SVR. The maximum error observed was 254.75 cm^2^/gr, while the average error was 74.42 cm^2^/gr. These results demonstrate that the SVR method outperforms the GB and RR approaches, as confirmed by the distribution of errors.

### 3.3. Model’s Comparison Using Statistical Performance Indicators

Table 8 presents the MAE, RMSE, MAPE, and R^2^ values obtained from the statistical evaluation of the developed machine learning models.

For CE estimation, the MAE values were 2.071 kWh/ton for GB, 1.657 kWh/ton for RR, and 0.878 kWh/ton for SVR. The MAPE values were 3.544% for GB, 2.702% for RR, and 1.541% for SVR. The RMSE values were 2.863 kWh/ton for GB, 2.695 kWh/ton for RR, and 1.175 kWh/ton for SVR. The highest R^2^ and the lowest MAE, MAPE, and RMSE values for CE estimation were achieved with the SVR method.

**Figure 10:**
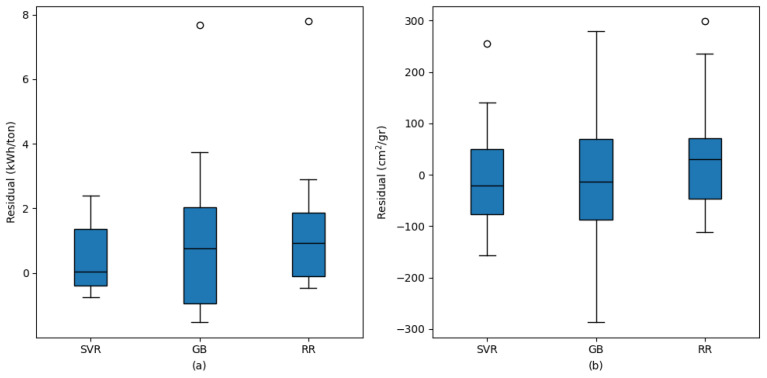
Residual comparison of built ML models for (**a**) CE and (**b**) BF values.

For BF estimation, the MAE values were 107.853 cm^2^/gr for GB, 77.068 cm^2^/gr for RR, and 74.421 cm^2^/gr for SVR. The MAPE values were 4.068% for GB, 2.848% for RR, and 2.738% for SVR. The RMSE values were 136.508 cm^2^/gr for GB, 98.028 cm^2^/gr for RR, and 89.929 cm^2^/gr for SVR. Similarly to CE estimation, the highest R^2^ and the lowest MAE, MAPE, and RMSE values for BF estimation were also obtained with the SVR method. Figure 10 presents the residual distributions of the models used for estimating CE and BF.

**Figure A1:**
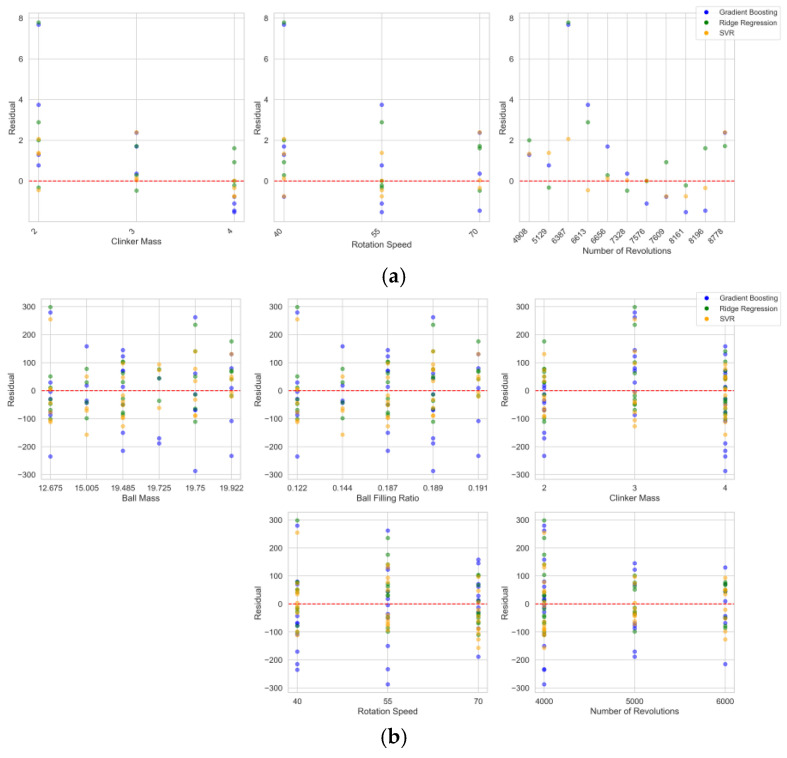
Residuals of (**a**) CE and (**b**) BF prediction across input variables for ML models.

Figure 10a,b illustrate the residual distributions of the models for CE and BF estimations, respectively. In both variables, the SVR model stands out as having the narrowest residual range and the most consistent error distribution in general, indicating predictions that are closer to the actual values with less variability. Conversely, the GB model exhibits the widest residual range and thus the highest error variance, reflecting greater deviations in its predictions. The RR model falls between GB and SVR, showing a moderate level of error spread. These graphical observations align with the MAE, MAPE, and RMSE values presented in the tables, reinforcing that SVR delivers superior performance for both CE and BF estimations. Furthermore, residuals with respect to individual input variables are provided in Appendix A Figure A1 for both CE and BF estimations, allowing interested readers to examine the model performances in more detail across different input conditions.

### 3.4. ML in Optimizing Cement Grinding Processes

It is noted that, among the machine learning methods utilized in this study, the SVR model yielded the highest R^2^ values, with values of 0.988 for CE prediction and 0.977 for BF prediction, thereby surpassing the performance of the GB and RR models. As a result of its superior predictive capability, the generalization performance of the SVR model was further evaluated using the test datasets that were not included in the training phase. For CE prediction, for instance, 11 previously unseen samples (i.e., samples that were not used to train the models) were employed, resulting in minimum, maximum, and average prediction errors of 0.02 kWh/ton, 2.39 kWh/ton, and 0.87 kWh/ton, respectively. Similarly, in BF prediction, 33 independent test samples were used, yielding the respective minimum, maximum and average errors of 3.56 cm^2^/gr, 254.75 cm^2^/gr, and 74.42 cm^2^/gr.

These findings clearly demonstrate that the corresponding ML model (i.e., SVR) possesses a strong capacity for generalization, delivering highly accurate predictions on previously unseen data and maintaining acceptable error margins. Moreover, it is also noted that the performance metrics of the other ML models utilized in the study were quite satisfactory. As a result, the ability to obtain such predictions without the need for additional experimental trials implies the potential of ML-based approaches to reduce experimental efforts. Consequently, well-trained ML models can serve as efficient tools for estimating CE and BF values corresponding to new process parameters, thereby facilitating the optimization of cement grinding operations and enhancing overall process efficiency.

## 4. Conclusions

In this study, the consumption of energy (CE) and Blaine fineness (BF) parameters were estimated using three different machine learning approaches, gradient boosting (GB), ridge regression (RR), and support vector regression (SVR), with clinker grinding conditions such as maximum ball size, ball filling ratio, clinker mass, rotation speed (rpm), and number of revolutions as input features. Initially, data preprocessing was performed, followed by univariate outlier analysis, where no outliers were detected. Feature selection was carried out using three methods: MIR, LR, and SBS. Based on the results of MIR and LR, the features selected for CE estimation were clinker mass, rotation speed, and number of revolutions. Similarly, for BF estimation, the feature selected was maximum ball size, along with ball mass, ball filling ratio, clinker mass, rotation speed, and number of revolutions.

Using the selected features, the GB, RR, and SVR methods were applied to estimate the CE and BF parameters. Adjustable hyperparameters and their ranges were determined for each method, and the models were evaluated using k-fold cross-validation (k = 10) with the ideal hyperparameters found through grid search. The performance of the applied models was assessed using evaluation metrics such as R^2^, MAE, MAPE, and RMSE. The results indicated that the machine learning methods were effective for estimating the relevant parameters. Specifically, SVR showed the best performance for both CE and BF estimations compared to the other approaches. Additionally, as shown in the Appendix A (Table A1 and Table A2), ensemble methods using simple average and weighted average approaches increased prediction accuracy, particularly improving results in BF estimation. Interested readers are encouraged to consult these tables for detailed results.

The experimental determination of optimal grinding parameters—such as clinker dosage, ball size distribution, and rotational speed—in ball milling processes is often time-consuming and resource-intensive. In this study, the application of machine learning techniques proved effective in identifying these optimal conditions, thereby reducing time, energy consumption, labor, and overall operational costs.

It is important to note that the models developed in this study are based on data collected under laboratory-scale grinding conditions. While these conditions offer controlled environments for model development, they may differ significantly from industrial-scale operations in terms of equipment size, process dynamics, and energy input. Therefore, direct application of the developed models to industrial settings may not yield equally accurate predictions.

Future studies could explore the use of transfer learning techniques, where models trained on laboratory data are fine-tuned with a small set of industrial data to improve generalizability. Alternatively, scaling factor analysis could be employed to mathematically bridge the differences between laboratory and industrial parameters, allowing for more reliable adaptation of the models in practical applications. Additionally, due to the limited number of observations in our dataset, future work may explore data augmentation techniques and Bayesian approaches.

## Figures and Tables

**Table 1:** Statistical parameters of the consumed energy data sample.

Parameter	Input Variables						Output
	Ball Mass	Maximum Ball Size	Ball Filling Ratio	Clinker Mass	Rotation Speed (rpm)	Number of Revolutions	Consumed Energy (kWh/ton)
Mean	17.76	58.67	0.17	3.00	55.00	7286.57	52.84
Standard Error	0.39	1.42	0.00	0.11	1.68	235.82	1.00
Median	19.61	65.00	0.19	3.00	55.00	7234.00	52.17
Mode	12.68	65.00	0.19	2.00	40.00	4510.00	38.40
Standard Deviation	2.88	10.45	0.03	0.82	12.36	1732.89	7.33
Range	7.25	28.00	0.07	2.00	30.00	7457.00	37.72
Minimum	12.68	37.00	0.12	2.00	40.00	4510.00	38.40
Maximum	19.92	65.00	0.19	4.00	70.00	11,967.00	76.12

**Table 2:** Statistical parameters of the Blaine fineness data sample.

Parameter	Input Variables						Output
	Ball Mass	Maximum Ball Size	Ball Filling Ratio	Clinker Mass	Rotation Speed (rpm)	Number of Revolutions	Blaine Fineness (cm^2^/gr)
Mean	17.76	58.67	0.17	3.00	55.00	5000.00	2994.69
Standard Error	0.23	0.82	0.00	0.06	0.97	64.35	48.70
Median	19.61	65.00	0.19	3.00	55.00	5000.00	3050.00
Mode	12.68	65.00	0.19	2.00	40.00	4000.00	2450.00
Standard Deviation	2.86	10.39	0.03	0.82	12.29	819.03	619.87
Range	7.25	28.00	0.07	2.00	30.00	2000.00	4021.00
Minimum	12.68	37.00	0.12	2.00	40.00	4000.00	289.00
Maximum	19.92	65.00	0.19	4.00	70.00	6000.00	4310.00

**Table 3:** Feature selection summary for CE and BF prediction.

	CE			BF		
Feature	MIR	LR	SBS	MIR	LR	SBS
Ball Mass	✗	✗	✓	✓	✗	✓
Maximum Ball Size	✗	✗	✗	✗	✓	✗
Ball Filling Ratio	✗	✗	✗	✓	✓	✓
Clinker Mass	✓	✓	✓	✓	✓	✓
Rotation Speed	✓	✓	✓	✓	✗	✓
Number of Revolutions	✓	✓	✗	✓	✓	✓

✗: Excluded feature, ✓: Selected feature.

**Table 4:** Hyperparameter settings for ML models in consumption of energy estimation.

Parameter	Gradient Boosting		Ridge Regression		Support Vector Regression	
	Range	Optimal Value	Range	Optimal Value	Range	Optimal Value
No. of estimator	10–300	200	-	-	-	-
Learning rate	0.01–1.0	0.5	-	-	-	-
Max. depth	1–5	1	-	-	-	-
Max. features	0.8–1.0	1.0	-	-	-	-
Min. sample leaf	1–4	1	-	-	-	-
Min. sample split	2–12	8	-	-	-	-
Alpha	-	-	0.001–100.0	0.1	-	-
Kernel	-	-	-	-	[RBF, linear]	RBF
C	-	-	-	-	0.1–100.0	100
Epsilon	-	-	-	-	0.01–0.5	0.5

**Table 5:** Hyperparameter settings for ML models in Blaine fineness estimation.

Parameter	Gradient Boosting		Ridge Regression		Support Vector Regression	
	Range	Optimal Value	Range	Optimal Value	Range	Optimal Value
No. of estimator	10–200	50	-	-	-	-
Learning rate	0.01–1.0	0.2	-	-	-	-
Max. depth	1–5	2	-	-	-	-
Max. features	0.8–1.0	0.9	-	-	-	-
Min. sample leaf	1–4	4	-	-	-	-
Min. sample split	2–12	6	-	-	-	-
Alpha	-	-	0.001–100.0	1	-	-
Kernel	-	-	-	-	[RBF, linear]	linear
C	-	-	-	-	0.1–100.0	100
Epsilon	-	-	-	-	0.01–0.5	0.01

**Table 6:** K-fold validation results for optimal hyperparameters in consumption of energy estimation.

Model	Parameter	K-Fold Number										Avg.
		1	2	3	4	5	6	7	8	9	10	
GB	MAE	0.79	2.70	1.02	2.52	1.75	2.03	1.30	1.33	0.52	1.33	1.53
	MAPE	1.53	5.23	2.04	5.08	2.51	3.82	2.39	2.45	1.06	2.76	2.89
	RMSE	1.02	3.27	1.23	3.31	3.07	2.27	1.55	1.54	0.60	1.86	1.97
	$R^{2}$	0.91	0.77	0.96	0.87	0.92	0.60	0.92	0.96	0.99	0.70	0.86
RR	MAE	1.88	1.98	1.29	1.88	2.57	0.86	2.11	1.03	1.75	2.28	1.76
	MAPE	3.64	3.92	2.54	3.33	3.82	1.57	3.80	2.15	3.80	4.49	3.31
	RMSE	1.90	2.44	1.48	2.26	3.75	1.14	2.21	1.23	2.02	2.46	2.09
	$R^{2}$	0.69	0.87	0.94	0.94	0.88	0.90	0.83	0.97	0.93	0.47	0.84
SVR	MAE	1.05	1.56	1.18	1.06	0.97	1.44	0.72	1.15	0.15	1.20	1.05
	MAPE	1.98	2.92	2.20	2.03	1.53	2.71	1.36	2.18	0.31	2.40	1.96
	RMSE	1.53	1.84	1.37	1.19	1.29	1.79	0.95	1.30	0.18	1.41	1.29
	$R^{2}$	0.80	0.93	0.95	0.98	0.99	0.75	0.97	0.97	1.00	0.83	0.92

**Table 7:** K-fold validation results for optimal hyperparameters in Blaine fineness estimation.

Model	Parameter	K-Fold Number										Avg.
		1	2	3	4	5	6	7	8	9	10	
GB	MAE	208.48	111.58	58.43	115.88	51.78	126.78	72.35	133.02	82.00	237.92	119.82
	MAPE	7.33	3.81	1.97	3.81	1.77	4.41	2.61	4.69	2.85	59.50	9.27
	RMSE	304.49	143.42	71.88	135.00	70.38	189.62	86.06	171.58	96.50	664.70	193.36
	$R^{2}$	0.82	0.91	0.98	0.95	0.99	0.86	0.97	0.93	0.96	0.46	0.88
RR	MAE	143.75	157.60	92.75	102.59	73.00	75.23	89.02	126.43	93.27	244.93	119.86
	MAPE	5.52	5.32	3.16	3.54	2.70	2.78	2.95	4.97	3.31	60.61	9.49
	RMSE	184.59	198.90	104.52	150.33	117.01	93.66	101.11	193.74	114.40	678.00	193.63
	$R^{2}$	0.94	0.82	0.96	0.94	0.96	0.97	0.96	0.90	0.94	0.43	0.88
SVR	MAE	127.11	136.37	86.32	98.74	77.27	69.78	80.43	142.63	86.00	251.11	115.58
	MAPE	4.90	4.65	2.88	3.40	2.80	2.59	2.79	5.55	3.09	59.70	9.24
	RMSE	174.17	168.68	98.19	141.83	116.06	91.01	87.70	199.78	104.39	665.32	184.71
	$R^{2}$	0.94	0.87	0.97	0.95	0.96	0.97	0.97	0.90	0.95	0.45	0.89

**Table 8:** Statistical test results for the built ML models.

	Consumption of Energy				Blaine Fineness			
Model	$R^{2}$	MAE (kWh/ton)	MAPE (%)	RMSE (kWh/ton)	$R^{2}$	MAE (cm^2^/gr)	MAPE (%)	RMSE (cm^2^/gr)
GB	0.9320	2.070	3.544	2.863	0.9469	107.853	4.068	136.508
RR	0.9396	1.657	2.702	2.695	0.9726	77.068	2.848	98.028
SVR	0.9885	0.878	1.541	1.175	0.9769	74.420	2.738	89.929

## Data Availability

The original contributions presented in this study are included in the article. Further inquiries can be directed to the corresponding author.

## Appendix A

**Table A1:** K-fold validation results for ensemble methods in consumption of energy estimation.

Model	Parameter	K-Fold Number										Avg.
		1	2	3	4	5	6	7	8	9	10	
Avg.	MAE	1.14	1.01	0.86	0.82	1.00	0.95	1.10	1.38	0.83	2.55	1.16
	MAPE	2.39	1.78	1.79	1.55	1.90	1.90	2.09	2.66	1.51	3.69	2.12
	RMSE	1.35	1.17	0.94	0.97	1.20	1.31	1.33	1.73	1.00	3.63	1.46
	$R^{2}$	0.96	0.95	0.97	0.96	0.87	0.97	0.90	0.76	0.97	0.92	0.92
WAvg.	MAE	0.77	0.77	0.64	0.88	0.98	0.82	0.99	1.56	0.83	2.14	1.04
	MAPE	1.56	1.37	1.32	1.67	1.87	1.62	1.87	3.01	1.47	3.15	1.89
	RMSE	0.86	0.91	0.78	0.99	1.29	1.21	1.35	1.86	1.12	3.02	1.34
	$R^{2}$	0.98	0.97	0.98	0.96	0.85	0.97	0.90	0.72	0.97	0.95	0.92

**Table A2:** K-fold validation results for ensemble methods in Blaine fineness estimation.

Model	Parameter	K-Fold Number										Avg.
		1	2	3	4	5	6	7	8	9	10	
Avg.	MAE	140.35	118.29	43.12	92.74	58.74	77.26	91.42	136.42	78.09	249.29	108.57
	MAPE	5.11	4.06	1.43	3.17	2.15	2.90	3.23	5.37	2.74	60.41	9.06
	RMSE	169.81	147.01	50.22	121.14	81.62	104.78	103.58	168.17	85.10	676.09	170.75
	$R^{2}$	0.95	0.90	0.99	0.96	0.98	0.96	0.96	0.93	0.97	0.44	0.90
WAvg.	MAE	143.78	119.89	38.07	88.36	52.19	66.77	82.77	129.01	73.23	245.18	103.93
	MAPE	5.06	4.03	1.28	3.02	1.93	2.45	2.98	5.01	2.55	60.14	8.85
	RMSE	178.03	151.96	46.62	114.71	74.71	93.67	91.53	158.24	83.70	673.10	166.63
	$R^{2}$	0.94	0.90	0.99	0.96	0.98	0.97	0.97	0.94	0.97	0.44	0.91

## References

[1] Touil D., Belaadi S., Frances C. (2008). The specific selection function effect on clinker grinding efficiency in a dry batch ball mill. Int. J. Miner. Process..

[2] ICS (2009). Cement Technology Roadmap 2009 Carbon Emission Reduction up to 2050.

[3] Qian H.Y., Kong Q.G., Zhang B.L. (2013). The effects of grinding media shapes on the grinding kinetics of cement clinker in ball mill. Powder Technol..

[4] Mardani-Aghabaglou A., İlhan M., Özen S. (2019). The effect of shrinkage reducing admixture and polypropylene fibers on drying shrinkage behaviour of concrete. Cem.-Wapno-Beton = Cem. Lime Concr..

[5] Kobya V., Kaya Y., Mardani-Aghabaglou A. (2022). Effect of amine and glycol-based grinding aids utilization rate on grinding efficiency and rheological properties of cementitious systems. J. Build. Eng..

[6] Sezer A., Boz A., Tanrinian N. (2016). An investigation into strength and permittivity of compacted sand-clay mixtures by partial replacement of water with lignosulfonate. Acta Phys. Pol. A.

[7] Yüksel C., Mardani-Aghabaglou A., Beglarigale A., Yazıcı H., Ramyar K., Andiç-Çakır Ö. (2016). Influence of water/powder ratio and powder type on alkali–silica reactivity and transport properties of self-consolidating concrete. Mater. Struct..

[8] Coppola L., Bellezze T., Belli A., Bignozzi M.C., Bolzoni F., Brenna A., Yang F. (2018). Binders alternative to Portland cement and waste management for sustainable construction—Part 1. J. Appl. Biomater. Funct. Mater..

[9] Coppola L., Bellezze T., Belli A., Bignozzi M.C., Bolzoni F., Brenna A., Yang F. (2018). Binders alternative to Portland cement and waste management for sustainable construction–Part 2. J. Appl. Biomater. Funct. Mater..

[10] Ahmad M.R., Chen B. (2020). Microstructural characterization of basalt fiber reinforced magnesium phosphate cement supplemented by silica fume. Constr. Build. Mater..

[11] Collivignarelli M.C., Abba A., Miino M.C., Cillari G., Ricciardi P. (2021). A review on alternative binders, admixtures and water for the production of sustainable concrete. J. Clean. Prod..

[12] Şahin H.G., Biricik Ö., Mardani-Aghabaglou A. (2022). Polycarboxylate-based water reducing admixture–clay compatibility; literature review. J. Polym. Res..

[13] Durgun M.Y., Özen S., Karakuzu K., Kobya V., Bayqra S.H., Mardani-Aghabaglou A. (2022). Effect of high temperature on polypropylene fiber-reinforced mortars containing colemanite wastes. Constr. Build. Mater..

[14] Liu Q., Tong T., Liu S., Yang D., Yu Q. (2014). Investigation of using hybrid recycled powder from demolished concrete solids and clay bricks as a pozzolanic supplement for cement. Constr. Build. Mater..

[15] Mardani-Aghabaglou A., Özen S., Altun M.G. (2018). Durability performance and dimensional stability of polypropylene fiber reinforced concrete. J. Green Build..

[16] Yiğit B., Salihoğlu G., Mardani-Aghabaglou A., Salihoğlu N.K., Özen S. (2020). Recycling of sewage sludge incineration ashes as construction material. J. Fac. Eng. Archit. Gazi Univ..

[17] Phillip E., Khoo K.S., Yusof M.A.W., Rahman R.A. (2022). Mechanistic insights into the dynamics of radionuclides retention in evolved POFA-OPC and OPC barriers in radioactive waste disposal. Chem. Eng. J..

[18] Lameck N.S., Kiangi K.K., Moys M.H. (2006). Effects of grinding media shapes on load behaviour and mill power in a dry ball mill. Miner. Eng..

[19] Erdem A.S., Ergün Ş.L. (2009). The effect of ball size on breakage rate parameter in a pilot scale ball mill. Miner. Eng..

[20] Shahbazi B., Jafari M., Parian M., Rosenkranz J., Chelgani S.C. (2020). Study on the impacts of media shapes on the performance of tumbling mills—A review. Miner. Eng..

[21] Abdelhaffez G.S., Ahmed A.A., Ahmed H.M. (2022). Effect of grinding media on the milling efficiency of a ball mill. Rud.-Geološko-Naft. Zb..

[22] Kaya Y., Kobya V., Mardani A., Mardani N., Beytekin H.E. (2024). Effect of Grinding Conditions on Clinker Grinding Efficiency: Ball Size, Mill Rotation Speed, and Feed Rate. Buildings.

[23] Altun O., Sert T., Altun D., Toprak A., Kwade A. (2024). Scale-up of Vertical Wet Stirred Media Mill (HIGmill) via Signature Plots, Stress Analyses and Energy Equations. Miner. Eng..

[24] Amiri S.H., Zare S. (2021). Influence of grinding and classification circuit on the performance of iron ore beneficiation–A plant scale study. Miner. Process. Extr. Metall. Rev..

[25] Dökme F., Güven O. (2014). Bilyalı değirmenlerde hızın performansa olan etkilerinin deneysel olarak incelenmesi. Mühendis Ve Makina.

[26] Mukhitdinov D., Kadirov Y., Boybutayev S., Boeva O., Babakhonova U. (2024). Simulation and control of ball mills under uncertainty conditions. J. Phys. Conf. Ser..

[27] Mavhungu E., Campos T.M., Rocha B.K.N., Solomon N., Bergmann C., Tavares L.M., Lichter J. (2024). Simulating large-diameter industrial ball mills from batch-grinding tests. Miner. Eng..

[28] Fortsch D.S. Ball charge loading-impact on specific power consumption and capacity. Proceedings of the IEEE Cement Industry Technical Conference, 2006, Conference Record.

[29] Göktaş İ., Altun O., Toprak N.A., Altun D. (2023). Element based ball mill and hydrocyclone modelling for a copper ore grinding circuit. Miner. Eng..

[30] Sridhar C.S., Sankar P.S., Prasad R.K. (2016). Grinding kinetics, modeling, and subsieve morphology of ball mill grinding for cement industry ingredients. Part. Sci. Technol..

[31] Altun D., Altun O., Zencirci S. (2019). Developing a methodology to model and predict the grinding performance of the dry stirred mill. Miner. Eng..

[32] Mardani-Aghabaglou A., Öztürk H.T., Kankal M., Ramyar K. (2021). Assessment and prediction of cement paste flow behavior; Marsh-funnel flow time and mini-slump values. Constr. Build. Mater..

[33] Feng D., Liu Z., Wang X., Chen Y., Chang J., Wei D., Jiang Z. (2020). Machine learning-based compressive strength prediction for concrete: An adaptive boosting approach. Constr. Build. Mater..

[34] Mustapha I.B., Abdulkareem M., Jassam T.M., AlAteah A.H., Al-Sodani K.A.A., Al-Tholaia M.M., Nabus H., Alih S.C., Abdulkareem Z., Ganiyu A. (2024). Comparative Analysis of Gradient-Boosting Ensembles for Estimation of Compressive Strength of Quaternary Blend Concrete. Int. J. Concr. Struct. Mater..

[35] Farooq F., Ahmed W., Akbar A., Aslam F., Alyousef R. (2021). Predictive modeling for sustainable high-performance concrete from industrial wastes: A comparison and optimization of models using ensemble learners. J. Clean. Prod..

[36] Marani A., Nehdi M. (2020). Machine learning prediction of compressive strength for phase change materials integrated cementitious composites. Constr. Build. Mater..

[37] Belalia Douma O., Boukhatem B., Ghrici M., Tagnit-Hamou A. (2017). Prediction of properties of self-compacting concrete containing fly ash using artificial neural network. Neural Comput. Appl..

[38] Yaman M.A., Abd Elaty M., Taman M. (2017). Predicting the ingredients of self compacting concrete using artificial neural network. Alex. Eng. J..

[39] Han Q., Gui C., Xu J., Lacidogna G. (2019). A generalized method to predict the compressive strength of high-performance concrete by improved random forest algorithm. Constr. Build. Mater..

[40] Zhang J., Ma G., Huang Y., Aslani F., Nener B. (2019). Modelling uniaxial compressive strength of lightweight self-compacting concrete using random forest regression. Constr. Build. Mater..

[41] Mai H., Nguyen T., Ly H., Tran V. (2021). Prediction Compressive Strength of Concrete Containing GGBFS using Random Forest Model. Adv. Civ. Eng..

[42] Farooq F., Amin M., Khan K., Sadiq M., Javed M., Aslam F., Alyousef R. (2020). A Comparative Study of Random Forest and Genetic Engineering Programming for the Prediction of Compressive Strength of High Strength Concrete (HSC). Appl. Sci..

[43] Iftikhar B., Alih S.C., Vafaei M., Elkotb M.A., Shutaywi M., Javed M.F., Deebani M., Khan I., Aslam F. (2022). Predictive modeling of compressive strength of sustainable rice husk ash concrete: Ensemble learner optimization and comparison. J. Clean. Prod..

[44] Sarir P., Chen J., Asteris P.G., Armaghani D.J., Tahir M.M. (2021). Developing GEP tree-based, neuro-swarm, and whale optimization models for evaluation of bearing capacity of concrete-filled steel tube columns. Eng. Comput..

[45] Shahmansouri A.A., Bengar H.A., Jahani E. (2019). Predicting compressive strength and electrical resistivity of eco-friendly concrete containing natural zeolite via GEP algorithm. Constr. Build. Mater..

[46] Aslam F., Farooq F., Amin M.N., Khan K., Waheed A., Akbar A., Javed M.F., Alyousef R., Alabdulijabbar H. (2020). Applications of gene expression programming for estimating compressive strength of high-strength concrete. Adv. Civ. Eng..

[47] Shah H.A., Rehman S.K.U., Javed M.F., Iftikhar Y. (2022). Prediction of compressive and splitting tensile strength of concrete with fly ash by using gene expression programming. Struct. Concr..

[48] Zeini H., Al-Jeznawi D., Imran H., Bernardo L., Al-Khafaji Z., Ostrowski K. (2023). Random Forest Algorithm for the Strength Prediction of Geopolymer Stabilized Clayey Soil. Sustainability.

[49] Zhou J., Su Z., Hosseini S., Tian Q., Lu Y., Luo H., Xu X., Huang J. (2024). Decision tree models for the estimation of geo-polymer concrete compressive strength. Math. Biosci. Eng..

[50] Chou J.S., Chiu C.K., Farfoura M., Al-Taharwa I. (2011). Optimizing the prediction accuracy of concrete compressive strength based on a comparison of data-mining techniques. J. Comput. Civ. Eng..

[51] Cheng M.Y., Cao M.T. (2016). Estimating strength of rubberized concrete using evolutionary multivariate adaptive regression splines. J. Civ. Eng. Manag..

[52] Kaveh A., Bakhshpoori T., Hamze-Ziabari S.M. (2018). M5′and Mars based prediction models for properties of self-compacting concrete containing fly ash. Period. Polytech. Civ. Eng..

[53] Nasr D., Babagoli R., Rezaei M., Andarz A. (2023). Evaluating the Influence of Carbon Fiber on the Mechanical Characteristics and Electrical Conductivity of Roller-Compacted Concrete Containing Waste Ceramic Aggregates Exposed to Freeze-Thaw Cycling. Adv. Mater. Sci. Eng..

[54] Cantu-Paz E. Feature Subset Selection, Class Separability, and Genetic Algorithms. Proceedings of the Genetic and evolutionary computation conference.

[55] Ladha L., Deepa T. (2011). Feature selection methods and algorithms. Int. J. Comput. Sci. Eng..

[56] Naqvi G. (2012). A Hybrid Filter-Wrapper Approach for Feature Selection. Master’s Thesis.

[57] Saeys Y., Inza I., Larranaga P. (2007). A review of feature selection techniques in bioinformatics. Bioinformatics.

[58] Bolon-Canedo V., Sanchez-Marono N., Alonso-Betanzos A., Benítez J.M., Herrera F. (2014). A review of microarray datasets and applied feature selection methods. Inf. Sci..

[59] Yang L., Shami A. (2020). On hyperparameter optimization of machine learning algorithms: Theory and practice. Neurocomputing.

[60] Singh S., Patro S.K., Parhi S.K. (2023). Evolutionary optimization of machine learning algorithm hyperparameters for strength prediction of high-performance concrete. Asian J. Civ. Eng..

[61] Thornton C., Hutter F., Hoos H.H., Leyton-Brown K. Auto-WEKA: Combined selection and hyperparameter optimization of classification algorithms. Proceedings of the 19th ACM SIGKDD International Conference on Knowledge Discovery and Data Mining.

[62] Kazemi F., Asgarkhani N., Jankowski R. (2023). Machine learning-based seismic fragility and seismic vulnerability assessment of reinforced concrete structures. Soil Dyn. Earthq. Eng..

[63] Nguyen H., Vu T., Vo T.P., Thai H.T. (2021). Efficient machine learning models for prediction of concrete strengths. Constr. Build. Mater..

[64] Touzani S., Granderson J., Fernandes S. (2018). Gradient boosting machine for modeling the energy consumption of commercial buildings. Energy Build..

[65] Marquardt D.W., Snee R.D. (1975). Ridge regression in practice. Am. Stat..

[66] Vapnik V. (1995). The Nature of Statistical Learning Theory.

[67] Drucker H., Burges C.J., Kaufman L., Smola A., Vapnik V. (1997). Support vector regression machines. Adv. Neural Inf. Process. Syst..

[68] Durgun H., İnce E.Y., İnce M., Çoban H.O., Eker M. (2023). Evaluation of Tree Diameter and Height Measurements in UAV Data through the Integration of Remote Sensing and Machine Learning Methods. Gazi J. Eng. Sci. (GJES)/Gazi Mühendislik Bilim. Derg..

[69] Ahmad A., Ahmad W., Aslam F., Joyklad P. (2022). Compressive strength prediction of fly ash-based geopolymer concrete via advanced machine learning techniques. Case Stud. Constr. Mater..

